# Establishment of fluoroscopy views and standardized procedure of percutaneous magic screw insertion for acetabulum fractures

**DOI:** 10.1186/s12891-018-2228-y

**Published:** 2018-09-12

**Authors:** Jiantao Li, Zhirui Li, Xiang Wang, Gongzi Zhang, Ye Peng, Shuwei Zhang, Peifu Tang, Lihai Zhang

**Affiliations:** Orthopedic Department, Chinese PLA General Hospital, Fuxing Road, 28, Beijing, 100853 People’s Republic of China

**Keywords:** Percutaneous magic screw, Acetabular fractures, 3D reconstruction model, Fluoroscopy views

## Abstract

**Background:**

To place the magic screw more simply, we established a set of reproducible fluoroscopic views and a standardized procedure of magic screw insertion.

**Materials and methods:**

This study on the magic screw tunnel uses a three-dimensional reconstruction model and a skeleton projection. The 3D model of the pelvis was made to be transparent and it was rotated to the place where the ischial spine was just sheltered by the posterior wall of the acetabulum. The angles of this view projection were recorded in the transverse plane and coronal plane. Six cadaveric pelvises (three males, three female) were used to validate the proper projection angle of the C-arm fluoroscopy. The skeleton specimens were all positioned latericumbent on a radiolucent table.

**Result:**

In all pelvis 3D models, all magic cylinders with a 7.3 mm diameter were successfully inserted along the bone structure tunnel in 30 3D pelvic models. The average angle of the transverse view rotated by the C-arm fluoroscopy was 162° in males and 157° in females, the angle of the coronal plane was 22° in males and 24° in females. The average distance between the front wheel of the C-arm machine and the middle axial line of the radiolucent bed was 43 cm in males and 43 cm in females. In skeleton pelvis research, all the screws were safely inserted using this method.

**Conclusion:**

The magic screw technique could be a good choice for the treatment of acetabular fractures, especially quadrilateral plate fractures. If the proper fluoroscopy view technique is used properly, the magic screw can be inserted rapidly and safely.

## Background

Acetabular fractures are characteristic injuries in high energy trauma patients, which remains to be the most challenging fracture for surgeons due to the complex anatomy and complicated injury mechanism [1]. The most used classification of acetabular fractures is the Letournel and Judet, which could properly guide the selection of treatment strategies [2].

Medial wall fractures of the acetabulum (quadrilateral plate) [3] are frequently encountered in fractures such as that of both column, anterior column and posterior hemitransverse, transverse, posterior column, or T-type fractures [4–7]. The fixation of the quadrilateral plate has been complicated and includes a risk of hip joint penetration [8, 9]. One percutaneous screw fixation technique, reported to hold the quadrilateral plate in a reduced position, was described as the “Magic screw” [10]. The effect of the magic screw to fix the quadrilateral plate can be the same as that of the posterior column screw, but is more convenient for body positioning, less invasive to the soft tissue, and has a higher strength to hold the quadrilateral area. However, the placement of the magic screw is technically demanding, even for an experienced orthopedic surgeon in our clinical practice [10, 11]. The optimal starting points, direction, and depth of a safe zone in the posterior column of the acetabulum and the accuracy of inserting these screws have always been controversial.

To place the magic screw more simply, a set of reproducible fluoroscopic views and a standardized procedure of magic screw insertion were established. This study delineates the technique of screw penetration based on anatomic studies, the readily reproducible fluoroscopic view, and the clinical practice in order to establish this new method.

## Materials and methods

All investigations were carried out in accordance with the ethical guidelines and were approved by the Institutional Ethical Review Committee of Chinese PLA General Hospital. All patients agree to use their data.

### The study on the magic screw tunnel using the three-dimensional reconstruction model

We collected the Digital Imaging and Communications in Medicine format (DICOM) of 30 patients and imported these into a personal computer. The DICOM data was extracted from 30 patients (15 male, 15 female, ages 26~ 82, height 155~ 183 cm, weight 48~ 80 kg) without deformity, fracture, or tumors who underwent computed tomography angiography in our hospital (Siemens Sensation Open 128-slice CT scanner; Siemens, Erlangen, Germany). Power settings were typically 100 kV, 105 mA, and 750 ms rotation time with a slice thickness of 1.2 mm. The field of view was 512 × 512 pixels with increments of 1.2 mm, using detector collimation of 128 × 0.625 mm (pitch 0.933). Then, the consecutive CT data were imported to the image-processing software of Mimics 15.0 (Materialise, Leuven, Belgium). All three-dimensional pelvis models were constructed through protocols including those for thresholding, region growing, masks editing, polylines calculation, and cavity filling from polylines.

**Fig. 1:**
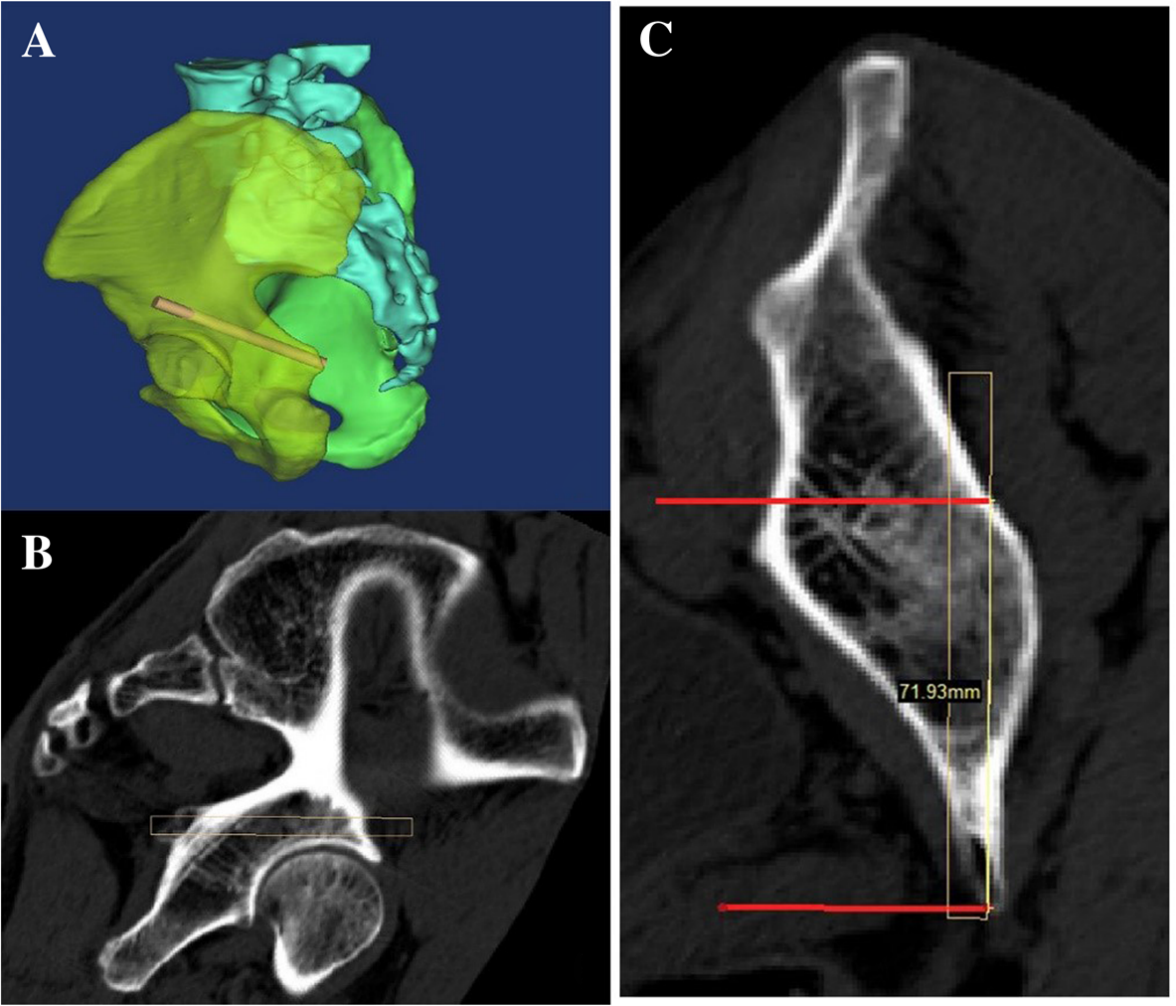
**a** The cylinder was walking along the bone channel behind the acetabular. **b**, **c** Through slightly adjusting the location of the cylinder, we confirmed that it was in the proper position without penetration into the hip joint

**Fig. 2:**
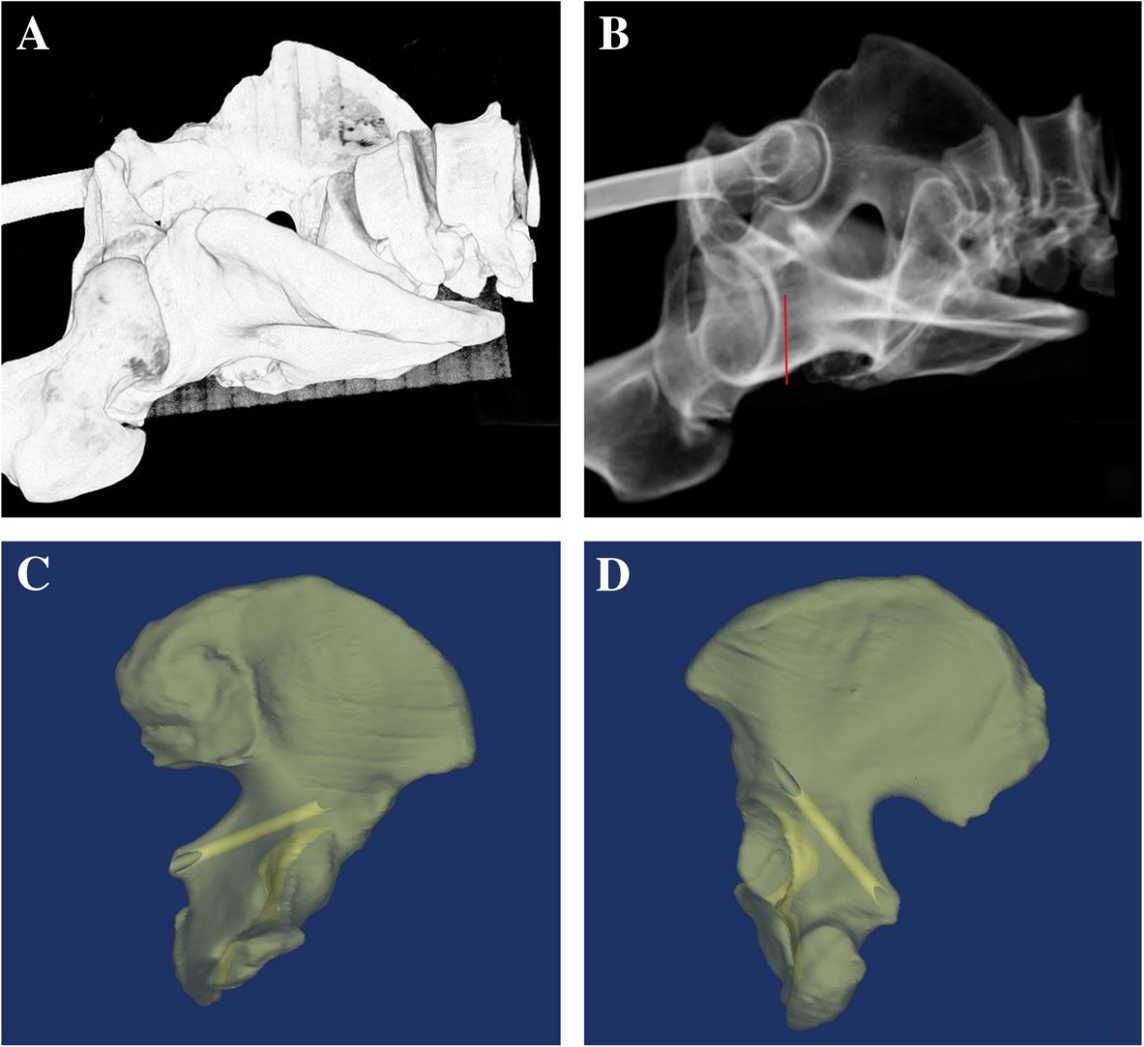
**a**, **b** The 3D model of the pelvis is shown and rotated to the right place where the ischial spine was just sheltered by the posterior wall of the acetabulum. The magic screw is manually depicted with the red line shown on picture B. **c**, **d** The magic screw channel is shown on the model

After the pelvis reconstruction, a cylinder with a 7.3 mm diameter was created with an average length of 73 mm (range 67–93 mm) using the function module of MedCAD in Mimics. One end side of the cylinder was placed on the lower part of the gluteus medius pillar, and the other end side was just medial to the ischial spine. The location of the cylinder was slightly adjusted to make it walk in the bone tunnel behind the acetabulum (Fig. 1). The 3D model of the pelvis was made to be transparent and it was rotated to the place where the ischial spine was just sheltered by the posterior wall of the acetabulum. The bone tunnel is shown in Fig. 2. The angles of this view projection were recorded in the transverse plane and coronal plane.

### The study on the skeleton projection

**Fig. 3:**
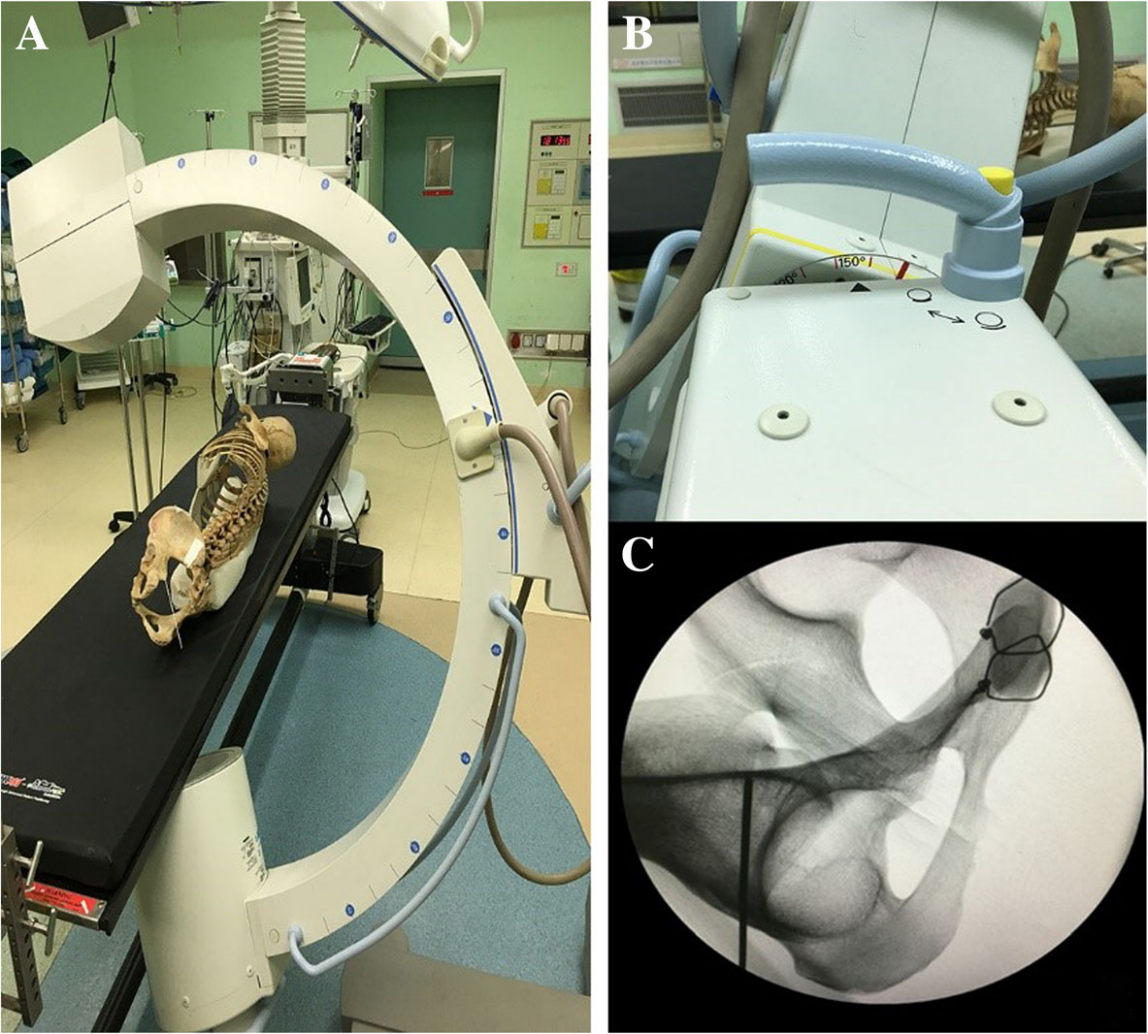
**a**, **b** The C-arm fluoroscopy was rotated to the proper angle. **c** We inserted the nail from the lower part of the gluteus medius pillar to the ischial spine under fluoroscopic image monitoring

Six cadaveric pelvises (three male, three female) were used to validate the proper projection angle of the C-arm fluoroscopy,which was performed in Beijing Aerospace General Hospital Discipline of Anatomy. The skeleton specimens were all positioned latericumbent on a radiolucent table. First, the standard lateral view of the pelvis was obtained, then the C-arm was rotated to the angles of the coronal and transverse plane. The images were recorded in every 5 degrees of rotation. When the proper image showed ischial spine overlapping with the posterior wall of the acetabulum, the position and rotation degree of C-arm fluoroscopy were recorded. Directed by fluoroscopic image, the starting point for the guide wire was 2 cm above the roof of the acetabulum on the gluteus medius pillar. The direction of the guide wire was to the ischial spine. With the starting point and the direction mentioned above, the guide wire was inserted and the magic screw was finally placed successfully (Fig. 3).

### Clinical application

Finally, a minimally displaced quadrilateral plate fracture was fixed with a percutaneous screw using this method. The patient was a 38 year old male, and the mechanism of the injury was a traffic accident. The fracture was on the left side. The time from injury to surgery was 4 days. According to OTA classification, the fracture model was 61-A2.3. The surgeon was a senior doctor with more than 10 years of experience in pelvis/acetabular cases. This case was performed using AP, inlet, and outlet plain radiographs after surgery.

The patient was placed on a radiolucent bed in the latericumbent position before general anesthesia, with the fracture side close to the edge of the bed. The axial view was verified by fluoroscopy and the magic screw technique was used. After the patient’s skin was circumferentially prepared and draped, the starting point of the body surface projection was found under the guidance of the lateral pelvis image. A 3 cm incision was made on the surface of the anterior column screw, which is on the gluteus medius pillar. After the entry-point was found, through rotating the fluoroscopy to the right place, the proper image was found as mentioned above. The 3.5 mm guide wire was placed into the bone percutaneously hand directed by the image. A mallet was used to gently insert the guide wire into the bone cortex (about 2 mm). A drill was not used because we feel that the mallet technique allows for better control. Under the C-arm fluoroscopic image, we could observe the guide wire crossing the fracture site, located at the exit-point of the ischial spine. After the placement of the wire and measurement of the depth, the screw was inserted with a 7.3 mm diameter and 75 mm length over the guide wire to fix the fracture. Final films included the iliac oblique, A-P, and obturator oblique views to ensure the screw was positioned at the right place without penetration in the hip joint.

## Results

In all pelvis 3D models, all magic cylinders with a 7.3 mm diameter were successfully inserted along the bone structure tunnel in 30 three-dimensional pelvic models. In skeleton pelvis research, all the screws were safely inserted using this method. The position and the rotation angle of the C-arm fluoroscopy were recorded in the skeleton specimen projection procedure. The average angle of the transverse view rotated by the C-arm fluoroscopy was 162° in males and 157° in females, the angle of the coronal plane was 22° in males and 24° in females. The average distance between the front wheel of the C-arm machine and the middle axial line of the radiolucent bed was 43 cm in males and 43 cm in females (Table 1).

**Table 1:** Angles for Males and Females in Axial View

Sex	Numbers	Transverse Plane, degrees	Coronal Plane, degrees	Distance (cm)
Male	1	157°	20°	45
	2	165°	25°	40
	3	165°	20°	43
Female	1	150°	25°	40
	2	157°	23°	45
	3	165°	25°	45

**Fig. 4:**
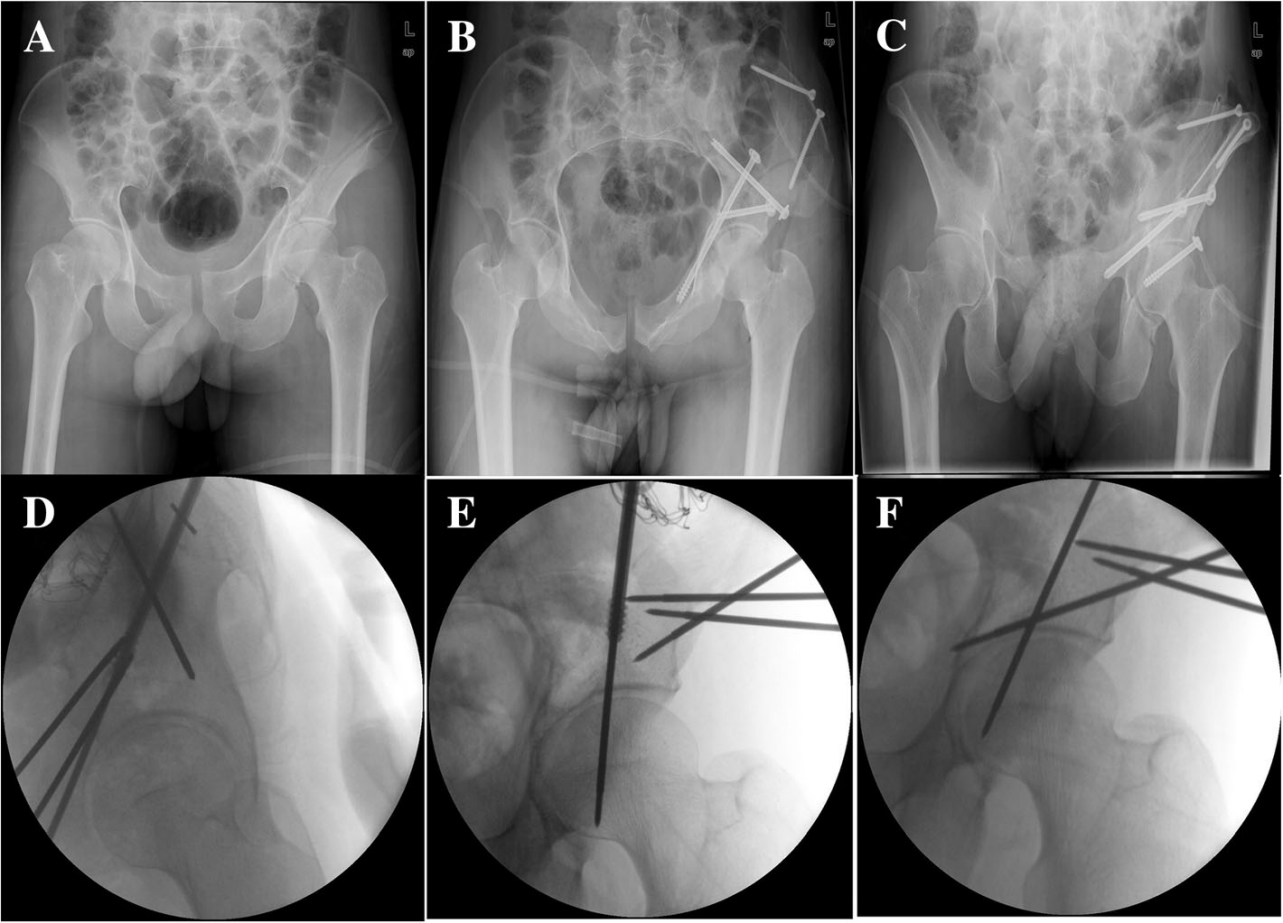
The standard procedure based on this technique. **a** Preoperative anteroposterior view of pelvic; **b** Postoperative inlet pelvic view; **c** Postoperative outlet pelvic view; **d** Intraoperative lateral views for placing K-ware; **e** and **f** intraoperative iliac oblique radiography view for placing K-ware

In clinical practice, the quadrilateral plate fractures of the patient were fixed by this method. The standard procedure based on this technique is shown in Fig. 4. The standard lateral image of magic screw projection can be achieved reproducibly during the surgery. Under this technique, the screw was inserted in a good position without penetration into the hip joint or vessel injury. The fluoroscopy time was 48 s, and the time of the magic screw fixation surgery was 40 min.

The patient was kept non-weight bearing for 8 weeks and advanced to weight bearing as tolerated at 2 months. This patient was followed-up at one year, and was healed. We did not observe any complications like venous thrombosis, screw breakage, reduction loss, dyspareunia, etc.

## Discussion

The magic screw is named as such because of its difficulty to shoot, and it is said that one needs supernatural powers to pass it [11]. The magic screw is used to hold the reduced quadrilateral plate as part of an initially displaced acetabular fracture. As it crosses the posterior column, it can also be used as an alternative to stabilize the quadrilateral fractures involving the posterior column [10]. The entry-point of the magic screw is similar to that of an anterior column screw, which is on the gluteus medius pillar, but near the roof of the acetabulum. The tunnel of this is behind the hip joint, directly down towards the posterior column and medially towards the ischial spine [12].

However, to our best knowledge, this is the first time that the methods and techniques of the magic screw from radiological and operative aspects have been described. Using the Mimics software, we found the location of the magic screw channel in 30 pelvic models, and of which the space within the bone structures channel was large enough to inset a 7.3 mm lag screw. We reconstructed every 3D pelvic model and used rotated angles to guide and manipulate the rotation of the C-arm fluoroscopy to a get proper projection image. The 1.2 mm thickness of DICOM data used in the research makes the 3-D pelvic models clear. Besides the digital research process, the skeleton study was adopted to verify the feasibility of the magic screw technique, through which we recorded the accurate angle and distance to locate the C-arm fluoroscopy in the right place. Both procedures mentioned previously were the foundation to guarantee the feasibility and security of the magic screw to be used in clinical practice.

The technique of percutaneous screw fixation used to fix the acetabular fracture was first described by Routt and Starr in the 1990s [13, 14]. In 2001, professor Starr introduced different kinds of techniques about the percutaneous screw fixation and firstly proposed the magic screw to fix the quadrilateral plate fractures [10]. However, he did not report any clinical trials about this technique. It was stated that on the iliac oblique view, the magic screw can be seen behind the hip joint, and it is above the joint on the AP view. Its path running in the bone structure channel can be followed on an obturator oblique view, and using a particular view, swinging between the obturator outlet and inlet views, it can be seen protruding [12]. However, it is difficult to insert the magic screw following the methods mentioned above because the message is not perceptible enough for the surgeons guided by the C-arm fluoroscopy to implant the screw.

Fluoroscopic views are always the foundation of the percutaneous screw techniques to treat the acetabular fractures. Due to the complicated morphology of the pelvic structure and the differing position of the patient, the appropriate fluoroscopic image is frequently unachievable during the surgery [15–17]. Operation time will be extended while finding the ideal image and the entry point [18], especially when the standard fluoroscopic view was not established. Recording the position and the rotation angle of the C-arm fluoroscopy preoperatively can facilitate the percutaneous techniques and reduce radiation time [13, 14]. In clinical practice, we use adhesive bandage as the mark sticking on the ground. The distance between the bandage and the middle axial line of the radiolucent bed was about 40 cm. The label indicates the location of front wheel of the C-arm. We rotate the C-arm to the angle of 160° and 20°, and sometimes move the C-arm forwards or backwards. By taking the pictures of the pelvis, we slightly adjust the angel of C-arm. It’s practical to get the proper picture by finding the proper angle without too much difficulty.

Through 3D model and skeleton research, we confirmed the proper angle of the projection view in the transverse and coronal planes, by which we insert the magic screw into the specimen successfully. A gender factor was found that affected the rotation angle of C-arm fluoroscopy. The mean distance between the front wheel of the C-arm machine and the middle axial line of the radiolucent bed was 43 cm. The distance did not change much in different samples. Guided by the angles and distance measured above, it was easy to find the proper radiologic image and facilitated the insertion of the magic screw, which passed through the bone channel safely and directly. In the clinical practice, we successfully inserted the magic screw with less radiation. Additionally, the 40-min fixation time was satisfied. The fracture healed without any complications.

In our experience, some important aspects should be mentioned: (1) The reslice project function in Mimics software was useful to rebuild the orthogonal planes depended on the inserted cylinder, through which we could check whether the 7.3 mm screw was properly in the safe zone without penetration. (2) It is better to place the patient close to the edge of the affected side on the operating bed since it allows for more convenient manipulation of the C-arm fluoroscopy. (3) A mallet was used instead of a drill to advance the guide wire within the bone channel because it is easier for us to control the mallet technique without violating the outer corridor of the channel. (4) It is better to keep the end of the screw located on the ischial spine, because shooting laterally endangers the sciatic nerve and medially affects the pelvic viscera.

## Conclusion

The magic screw technique could be a good choice for the treatment of acetabular fractures, especially quadrilateral plate fractures. If the proper fluoroscopy view technique is used properly, the magic screw can be inserted rapidly and safely. However, our study has some limitations. All the CT data of the patients are from Chinese records, which may limit extrapolation of the results to other nationalities. The number of cases in our study is relatively small. Finally, there is no control group with which to compare the clinical results. More prospective randomized controlled trials are needed to overcome the limitations of our research.

## Abbreviations

3D: Three dimensional
CT: Computed tomography
DICOM: Digital Imaging and Communications in Medicine format
